# A Case of Peripartum Spontaneous Coronary Artery Dissection in a Woman With a History of Obesity

**DOI:** 10.7759/cureus.33021

**Published:** 2022-12-27

**Authors:** Taqi A Rizvi, Shahkar Khan, Ahmad Mustafa, Rabih Tabet, James C Lafferty

**Affiliations:** 1 Internal Medicine, Staten Island University Hospital, Staten Island, USA; 2 Cardiology, Staten Island University Hospital, Staten Island, USA

**Keywords:** post-partum, bariatric surgery, acs, obesity, scad

## Abstract

Spontaneous coronary artery dissection (SCAD) is a rare cause of acute coronary syndrome (ACS) with a high prevalence in young pregnant females. A 38-year-old female with a history of morbid obesity status post-bariatric surgery presented with chest pain. The electrocardiogram (EKG) revealed ST-segment elevation in the inferior leads as well as slightly elevated troponin. Urgent cardiac catheterization showed SCAD, and she was subsequently managed with medical therapy. We hypothesize that the history of obesity leads to a compromise in the coronary vasculature, thereby predisposing the patient to SCAD.

## Introduction

Spontaneous coronary artery dissection (SCAD) is a rare cause of acute coronary syndrome (ACS) [1-3]. It was formerly believed that SCAD classically affected young females without typical atherosclerotic risk factors; however, recent studies have demonstrated that the epidemiology is widespread and includes older patients with concomitant coronary artery disease (CAD) risk factors [3]. Nonetheless, it has been found to be the most common cause of pregnancy-associated myocardial infarction (PAMI) [1,4]. There have been studies to identify the association between pregnancy-related myocardial infarctions and traditional risk factors for ACS. However, the literature on the incidence of SCAD in pregnancy in the presence of traditional risk factors for CAD is sparse.

It has been hypothesized that hormonal and hemodynamic changes during pregnancy predispose patients to SCAD [4-6]. We present the case of a 38-year-old female with a history of previously documented obesity status post-bariatric surgery who was diagnosed with SCAD after a three-month post-partum period. We believe that their history of obesity predisposed her to SCAD.

## Case presentation

A 38-year-old female with three prior pregnancies, two prior childbirths, and a history of morbid obesity (BMI 42.2) status post-bariatric surgery approximately six years ago presented to the emergency department with the chief complaint of chest pain three months post-partum after a normal vaginal delivery. She reported having intermittent, pressure-like substernal pain for the last two days. The pain worsened an hour before her presentation and prompted her to go to the emergency department. In the emergency department, the pain was 8/10 in intensity, with radiation to the left arm. She denied any other complaints, including dizziness, diaphoresis, and palpitations. She denied any family history of coronary artery disease.

**Figure 1:**
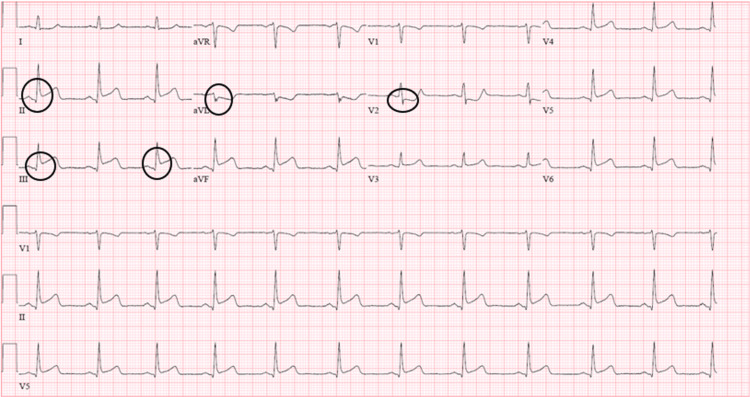
Encircled are ST-segment elevations in leads II, III, aVF, and ST depressions in aVL and V2.

**Figure 2:**
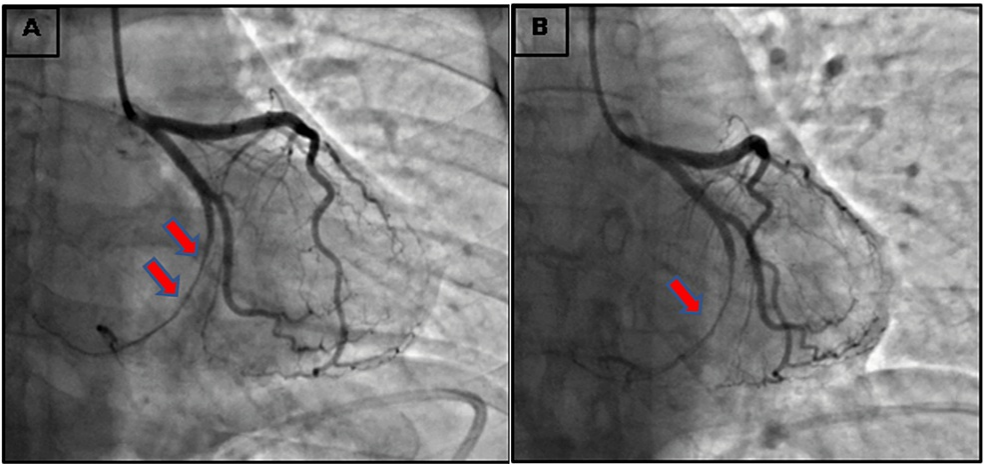
Pictures taken during cardiac angiogram showing SCAD of the left circumflex artery, represented by red arrows in pictures in (A) and (B).

Upon initial presentation, vital signs included a blood pressure of 108/62 mmHg, a heart rate of 74 beats per minute, a temperature of 99 degrees Fahrenheit, and an oxygen saturation of 98% on room air. A cardiovascular physical exam did not reveal any murmurs. Initial blood work revealed a white cell count of 11.51 K/µL, hemoglobin of 11.5 g/dl, troponin of 0.02 ng/ml, serum pro-brain natriuretic peptide of 53 pg/ml, high-density lipid of 71 mg/dl, low-density lipid of 77 mg/dl, and lactate of 0.6 mmol/L. The electrocardiogram showed ST segment elevation in leads II, III, and aVF and ST depressions in aVL and V2, as shown in Figure 1. The X-ray of the chest was unremarkable. She was taken for immediate coronary catheterization. The angiography findings were consistent with SCAD of the distal left circumflex artery, shown in Figure 2. Contrast ventriculography revealed a calculated ejection fraction (EF) of 50%.

**Figure 3:**
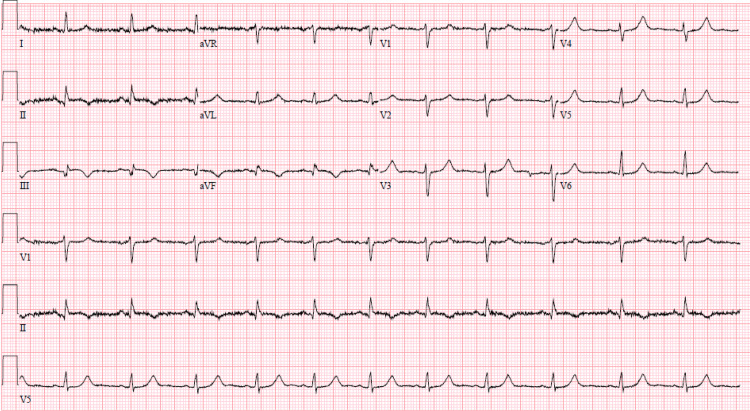
Resolution of ST segment changes

No coronary intervention was done, and she was treated with medical therapy, including dual antiplatelet therapy, a statin, and a low-dose beta-blocker. Serial cardiac enzymes were done, which showed troponins peaking at 1.80, which subsequently down-trended. Serial EKGs showed the resolution of ST segment changes, as shown in Figure 3. The patient was discharged on the same medications as prescribed during the hospital stay, with instructions for a close follow-up with her cardiologist. On her outpatient follow-up, she denied any subsequent episodes of chest pain.

## Discussion

SCAD mostly affects females, and one-third of these cases are present during the peripartum period. The initial presentation of SCAD is similar to other acute coronary syndromes [7,8]. Therefore, a high level of suspicion is required to diagnose SCAD, and it should be high on the differential diagnosis when a relatively young female presents with typical chest pain, especially in the peripartum period [2].

The risk factors associated with SCAD include, but are not limited to, atherosclerosis and the peripartum period. The most affected artery in SCAD is the left anterior descending (LAD) artery [2]. It is proposed that the hemodynamic changes that take place during pregnancy, including an increase in plasma volume and cardiac output, lead to increased shearing forces on the tunica intima and increase the probability of developing intimal tears. In addition, the decreased production of collagen and increased progesterone production lead to the weakening of the tunica media [4,7].

Obesity contributes more to the development of CAD in females than in males, as shown by the Farmingham Heart Study, in which the authors were able to show an increased attributable risk of hypertension and cardiovascular disease [9].

A study published by Chacin-Suarez et al. showed that 70% of the patients with SCAD included in the study had one or more cardiovascular risk factors. In this study, obesity and being overweight were found to be the most common risk factors [10].

One retrospective study by Clare et al. studied baseline characteristics of patients with SCAD and long-term outcomes and concluded that the improved survival of patients with SCAD compared to those without can be explained by favorable baseline characteristics, including fewer atherosclerotic risk factors. The study also highlights the lack of evidence regarding medical therapies and raises the important question of whether medications should be tailored according to the risk factors [11].

According to current literature, obesity is associated with a chronic inflammatory state resulting in vascular inflammation [5,6]. Obesity leads to the downregulation of vascular nitric oxide production and excessive reactive oxygen species formation, leading to vascular inflammation and dysfunction [7]. The authors hypothesize that a history of obesity leads to a compromise in the coronary vasculature, hence predisposing the presented patient to SCAD.

Coronary artery dissection can be classified based on etiology or based on angiographic findings. Based on etiology, it is divided into primary and secondary. Primary coronary artery dissections occur spontaneously, whereas secondary dissections are either due to extension from an aortic dissection, trauma, or secondary to percutaneous coronary intervention (PCI) and/or coronary artery bypass graft (CABG) surgery [12].

The best diagnostic modality for SCAD is coronary angiography. SAW classification is the most commonly used method to classify SCAD on the basis of angiographic findings. In type 1, a radiolucent flap can be seen angiographically with a linear double lumen, which likely represents a slower contrast transit time within the false lumen as compared to the true lumen. Type 2 generally presents as a long, smooth narrowing located at the mid-to-distal section of the artery, which can then be further divided into type 2a and type 2b. In type 2a, there is a normal-appearing vessel at the end of the stenosis, and in type 2b, the stenosis extends all the way to the end of the affected vessel. Type 3 SCAD lesions have visual similarities during angiography compared to focal atherosclerotic stenosis, which then further needs to be evaluated using intra-coronary imaging to distinguish between atherosclerotic stenosis and stenosis due to SCAD. Type 4 is classified as a total occlusion of a vessel, most commonly a distal vessel. Out of the SAW classifications, the most common lesions are those of type 2, followed by type 1 [12]. Angiography on our patient showed evidence of a type 2b lesion.

SCAD has been managed with conservative medical therapy, PCIs, and coronary artery bypass grafting; however, the most optimum treatment modality is still unclear. The American College of Cardiology recommends conservative treatment if the coronary blood flow is preserved and/or there is negligible ongoing ischemia with relative distal involvement of the coronary vessel. The Canadian SCAD cohort study reports that conservative management is the preferred treatment method unless high-risk features like left main dissection, ongoing ischemia, peripartum SCAD, and hemodynamic/electrical instability are present. PCIs have been shown to have a higher risk of complications and lower success rates as compared to atherosclerotic diseases. The success rates of PCI were reported in the Canadian SCAD cohort study, in which 103 PCI cases were performed for SCAD, of which only 29.1% were successful and 40.8% were deemed partially successful [13]. Due to the risk of recurrence of SCAD, another pregnancy is discouraged in such patients [14].

In addition, the use of intravascular ultrasound in conjunction with coronary angiography can help the provider not only with the diagnosis but also with manipulating the wires in the occluded vessel for interventions if indicated [15]. However, extreme care must be taken as coronary manipulation has been associated with an increased risk of dissection propagation [2].

It is noteworthy to mention that, due to the paucity of data on the management of SCAD, the choice between conservative and invasive treatment should be guided not only by evidence-based medicine but also by the clinical judgment of a multidisciplinary team that includes a cardiologist, a cardiothoracic surgeon, and, in some cases, an intensivist. In our case, the patient underwent conservative management as decided by a team of cardiologists and cardiothoracic surgeons and had a favorable outcome.

To the best of our knowledge, there are no studies with statistically significant outcomes that have studied the effects of traditional risk factors on SCAD. Further studies in this area will help establish guidelines for treatment options as well. In our presented case report, we speculate that traditional factors can potentially be independent risk factors for predisposition to SCAD.

## Conclusions

In conclusion, there is a need to conduct further studies to investigate the impact of traditional risk factors for CAD on SCAD, such as obesity. This will aid in the early recognition of high-risk patients, which can help in implementing preventive strategies to reduce morbidity and mortality due to SCAD. Our case presentation highlights obesity as a risk factor that may have contributed to SCAD in our patient.
